# Identifying Ordinal Similarities at Different Temporal Scales

**DOI:** 10.3390/e26121016

**Published:** 2024-11-24

**Authors:** Luciano Zunino, Xavier Porte, Miguel C. Soriano

**Affiliations:** 1 Centro de Investigaciones Ópticas (CONICET La Plata-CIC-UNLP), Gonnet 1897, La Plata, Argentina; lucianoz@ciop.unlp.edu.ar; 2 Departamento de Ciencias Básicas, Facultad de Ingeniería, Universidad Nacional de La Plata (UNLP), La Plata 1900, Argentina; 3 Institute of Photonics, Department of Physics, University of Strathclyde, 99 George Street, Glasgow G1 1RD, UK; javier.porte-parera@strath.ac.uk; 4 Instituto de Física Interdisciplinar y Sistemas Complejos (IFISC, UIB-CSIC), Campus Universitat de les Illes Balears, E-07122 Palma de Mallorca, Spain

**Keywords:** time series, symbolic analysis, ordinal patterns, permutation entropy, Jensen–Shannon divergence, permutation Jensen–Shannon distance, multiscale analysis, ordinal similarity, chaotic semiconductor laser, delayed optical feedback

## Abstract

This study implements the permutation Jensen–Shannon distance as a metric for discerning ordinal patterns and similarities across multiple temporal scales in time series data. Initially, we present a numerically controlled analysis to validate the multiscale capabilities of this method. Subsequently, we apply our methodology to a complex photonic system, showcasing its practical utility in a real-world scenario. Our findings suggest that this approach is a powerful tool for identifying the precise temporal scales at which two distinct time series exhibit ordinal similarity. Given its robustness, we anticipate that this method could be widely applicable across various scientific disciplines, offering a new lens through which to analyze time series data.

## 1. Introduction

It is widely known that quantifying the similarity (or dissimilarity) between two time series is an essential task for clustering and classification purposes [1]. Because of this main reason, a lot of methodologies have been developed to determine how much an arbitrary time series resembles another one. Actually, this issue can be addressed from very different perspectives: there are shape-, edit-, feature-, and structure-based measures [2]. Some of them are more computationally inspired, while others are based on physical notions. To the best of our knowledge, up to now, there is no optimal algorithm for estimating this concept in practice. It has also been demonstrated that the performance of similarity quantifiers can be highly reduced when time series with different sampling frequencies are contrasted [3,4], when there exist nonlinear dependencies between them [5] and/or in the presence of outliers [6]. Hence, a measure able to deal robustly with different types of data is sought.

The permutation Jensen-Shannon distance (PJSD) has recently been proposed within this realm [7]. It is a versatile and conceptually simple ordinal metric tool that, thanks to its noise robustness and invariance under scaling of the data, is particularly suited for the analysis of real-world signals [8]. The PJSD takes advantage of the Jensen-Shannon divergence (JSD) [9], a widely accepted method for assessing the dissimilarity between two probability distributions, and of the ordinal coarse-grained representation introduced more than 20 years ago by Bandt and Pompe (BP) [10]. The flexibility of the JSD to different distributional data types, together with the proven efficiency of the ordinal patterns for identifying equivalent dynamics [11] and for time series clustering [12], allow us to conjecture that the proposed fusion represents a useful addition to the repertoire of approaches intended to estimate the degree of similarity between two arbitrary time series.

In this work, we put special focus on the PJSD’s ability to test similarity between two time series at different time scales. Numerical and experimental analyses are included to illustrate this fact. The results obtained confirm that the PJSD robustly identifies the time scales that maximize the similarity between two arbitrary signals. Consequently, this ordinal metric, implemented through a multiscale scheme, offers an efficient alternative to characterize how similar two signals recorded at different sampling rates are. Taking into account that widely implemented similarity measures, like dynamic time warping, are strongly affected when facing this challenge [3,4], we consider our finding relevant enough and of potential interest for the time series analysis community.

The rest of this paper is structured as follows. A brief presentation of the PJSD is first given in Section 2. Then, in Section 3, a numerically controlled analysis is developed to illustrate how the multiscale PJSD approach works. After that, a more complex practical application comparing time series of semiconductor laser experiments is performed in Section 4. The main conclusions obtained from this study are finally summarized in the last Section 5.

## 2. Permutation Jensen–Shannon Distance

The PJSD can be estimated by calculating the square root of the normalized JSD between the ordinal probability distributions associated with the two time series under comparison [7]. Defined in such a way, it is a metric able to quantify the degree of discernability between two arbitrary time series from an ordinal perspective.

The JSD is a measure of the distance between two arbitrary probability distributions, $P=\{p_{1},\ldots ,p_{n}\}$ and $Q=\{q_{1},\ldots ,q_{n}\}$, given by

(1) $$\mathrm{JSD}\left(P,Q\right)=S\left(\frac{P+Q}{2}\right)-\frac{1}{2}S\left(P\right)-\frac{1}{2}S\left(Q\right)\;,$$

where *S* is the Shannon entropy function $S\left(P\right)=-\sum_{i=1}^{n}p_{i}\ln p_{i}$, and, as usual, the convention 0ln0=0 is assumed in accordance with its mathematical limit. The JSD is always a well-defined and bounded quantity [9] that achieves its minimum possible value, i.e., 0, when identical probability distributions are compared, while its maximum potential value, i.e., ln2, is obtained whenever the supports of *P* and *Q* are disjoints (that is, $p_{i}q_{i}=0$ for i=1,…,n). It has also been shown that ${\left[\mathrm{JSD}(P,Q)\right]}^{1/2}$ satisfies all the formal properties needed to be a metric [13]. Further statistical properties and theoretical interpretations of the JSD can be found in Ref. [14].

The estimation of the Jensen–Shannon distance, ${\left[\mathrm{JSD}(P,Q)\right]}^{1/2}$, between two time series requires us first to know the corresponding probability distributions, *P* and *Q*, associated with the two time series under analysis. This task is not straightforward nor simple, and it deserves careful attention [15]. We address it by implementing the BP mapping method. BP propose mapping a continuous-valued time series into a discrete series of ordinal symbols or ordinal patterns. Perhaps the most relevant property related to this symbolization scheme is the fact that, as stated by Amigó et al. [16], “ordinal patterns are not symbols ad hoc but they actually encapsulate qualitative information about the temporal structure of the underlying data”. That is, the presence of underlying temporal correlations in the dynamics of the process that generates the time series is naturally considered in the BP recipe. Next, we will briefly summarize the discretization of time series via ordinal patterns. For further technical details interested readers are referred to the reviews [16,17,18,19,20]. Given a real-valued time series $X=\{x_{t}\in R,t=1,\ldots ,L\}$, vectors of equally spaced *D* values of the form $\left(x_{s},x_{s+\tau },\ldots ,x_{s+(D-1)\tau }\right)$ with $s=1,\ldots ,L^{*}=L-\left(D-1\right)\tau$ are mapped to one of the D! possible ordinal permutations of the same size that describe the order relation between these elements. For example, the vector (2.5,4.7,0.6) is mapped to the ordinal or permutation pattern (2,3,1), replacing each element in the original vector with its respective ranking in the subset. Assigning a symbol $\pi_{i}$ to each ordinal pattern, the original time series is mapped to the coarse-graining sequence $Y=\{y_{s}\in \Pi_{D}\in ,s=1,\ldots ,L^{*}\}$, with $\Pi_{D}=\left\{\pi_{1},\pi_{2},\ldots ,\pi_{D!}\right\}$ representing the set of permutations of length *D*. Just for illustrative purposes, $\Pi_{2}=\left\{\pi_{1}=\left(1,2\right),\pi_{2}=\left(2,1\right)\right\}$, and $\Pi_{3}=\left\{\pi_{1}=\left(1,2,3\right),\pi_{2}=\left(1,3,2\right),\pi_{3}=\left(2,1,3\right),\pi_{4}=\left(2,3,1\right),\pi_{5}=\left(3,1,2\right),\pi_{6}=\left(3,2,1\right)\right\}$. Estimating the probability of each ordinal pattern $p(\pi_{i})$ based on its relative frequency of occurrence in the symbolized sequence, an associated ordinal probability distribution (OPD) can be then obtained as follows:

(2) $$P_{\pi }^{D,\tau }=\left\{p\left(\pi_{i}\right),i=1,\ldots ,D!\right\}.$$

Two parameters have to be fixed: the number of elements in the permutation patterns *D* (called the order or embedding dimension, D≥2 with D∈N) and the time separation τ between the elements in the subsequence (called lag or embedding delay, τ∈N). Consecutive data are considered if τ=1, while τ−spaced data samples are analyzed if τ≥2. A toy example is included below to illustrate the role played by the lag τ. Given the short time series X={1.1,2.1,4.3,3.2,6.7,0.5,10.4,8.9} and fixing D=3 and τ=2, the first vector $\left(x_{1},x_{3},x_{5}\right)=\left(1.1,4.3,6.7\right)$ is mapped to the ordinal pattern $\pi_{1}=\left(1,2,3\right)$. The second three-dimensional vector is $\left(x_{2},x_{4},x_{6}\right)=\left(2.1,3.2,0.5\right)$, and $\pi_{4}=\left(2,3,1\right)$ is its associated ordinal pattern. Finally, the following two remaining vectors, $\left(x_{3},x_{5},x_{7}\right)=\left(4.3,6.7,10.4\right)$ and $\left(x_{4},x_{6},x_{8}\right)=\left(3.2,0.5,8.9\right)$, are mapped to the permutations $\pi_{1}=\left(1,2,3\right)$ and $\pi_{3}=\left(2,1,3\right)$, respectively. Consequently, the symbolic sequence $Y=\{\pi_{1},\pi_{4},\pi_{1},\pi_{3}\}$ is obtained when applying the BP coarse-graining procedure with parameters D=3 and τ=2, and the OPD turns out to be $P_{\pi }^{3,2}=\{p\left(\pi_{1}\right)=0.5,p\left(\pi_{2}\right)=0,p\left(\pi_{3}\right)=0.25,p\left(\pi_{4}\right)=0.25,p\left(\pi_{5}\right)=0$, $p(\pi_{6})=0\}$ for this simple numerical example.

On the one hand, the condition L≫D!, with *L* representing the number of data in the original time series, is required for a robust estimation of $P_{\pi }^{D,\tau }$. It is also clear that larger values of *D* offer improved characterization of the system dynamics. Actually, the order *D* has to exceed a lower bound $D_{min}$ to successfully resolve the underlying temporal structures for data from high-dimensional systems [21]. On the other hand, a value of the lag τ=1 is often used in discrete systems, and also when the chosen sampling frequency is the optimal one to characterize the underlying dynamics of continuous systems [18]. However, this arbitrary choice can lead to erroneous conclusions, especially for systems with scale-dependent dynamics [22]. A multiscale analysis, by analyzing how descriptors of the OPD change with τ, gives a more complete picture in these instances [23,24,25], also providing a practical and efficient alternative for identifying time delays from stochastic and chaotic models [26,27].

Among the different statistics that can be computed from the resulting OPD given by Equation (2), the permutation entropy [10] is undoubtedly the most representative and widely implemented quantifier. Defined as the Shannon entropy of this ordinal distribution, $S\left(P_{\pi }^{D,\tau }\right)=-\sum_{i=1}^{D!}p\left(\pi_{i}\right)\log p\left(\pi_{i}\right)$, it quantifies the variety of permutation patterns in the ordinal sequence obtained from a time series. However, many other much more complex descriptors of the OPD, which try to characterize some particular aspect of it, have been proposed. Without being exhaustive, we can mention permutation statistical complexity [28], Rényi permutation entropy [29] and permutation Fisher’s information measure [30].

The PJSD is defined as the normalized Jensen–Shannon distance between the OPDs, $P_{\pi }^{D,\tau_{1}}$ and $Q_{\pi }^{D,\tau_{2}}$, associated with two arbitrary time series, i.e.,

(3) $${\left[\mathrm{JSD}\left(P_{\pi }^{D,\tau_{1}},Q_{\pi }^{D,\tau_{2}}\right)/\ln\left(2\right)\right]}^{1/2}.$$

The ordinal mapping of different time series is compared using the PJSD. It is worthy to highlight here that, when estimating the PJSD, the order *D* chosen to implement the BP symbolization recipe should be the same for both time series in order to have the same number of possible permutation patterns in the OPDs to be compared. However, different lags, $\tau_{1}$ and $\tau_{2}$, can be selected, opening the possibility of contrasting the ordinal similarity of only one or two time series at two different temporal scales. The potentialities of this multiscale approach are explored in this article.

## 3. An Illustrative Numerical Example

We focus our analysis on nonlinear systems in the chaotic regime, where the dynamics involve multiple time scales. In particular, we use the chaotic Mackey–Glass (MG) system as an example, which is a paradigmatic case of a high-dimensional (strange) chaotic attractor [31,32]. The evolution of the MG system, denoted by x(t), is described by the following delay differential equation:

(4) $$\dot{x}\left(t\right)=-ax\left(t\right)+\frac{bx(t-\tau_{S})}{1+x{\left(t-\tau_{S}\right)}^{c}},$$

where a=0.1, b=0.2 and c=10 are standard parameters [32], and the delay $\tau_{S}=30$ sets the system into the chaotic regime [32,33].

**Figure 1:**
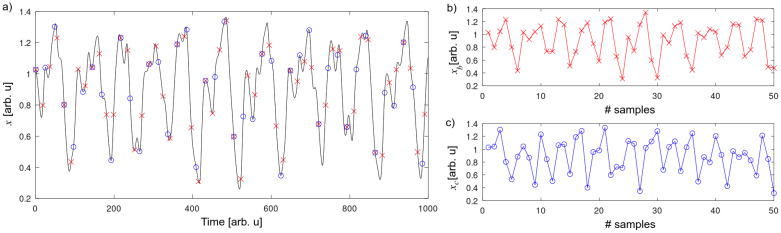
(**a**) Time series of MG system. Red crosses indicate sampling of $t_{b}=18\cdot 10^{2}\Delta t$ and blue empty circles sampling of $t_{c}=24\cdot 10^{2}\Delta t$. (**b**) Example of sequence extracted with sampling $t_{b}$. (**c**) Example of sequence extracted with sampling $t_{c}$.

We numerically integrate Equation (4) with an integration step of Δt=0.01, generating long time series, which are subsequently sampled for our analysis. Figure 1a presents a time series of the MG system in the chaotic regime, where we indicate different sampling intervals with either crosses or empty circles. The corresponding subsampled time series are shown in Figure 1b,c, where two different sampling intervals, $t_{b}=18\cdot 10^{2}\Delta t$ (crosses, $x_{b}$) and $t_{c}=24\cdot 10^{2}\Delta t$ (circles, $x_{c}$), are chosen for illustrative purposes. As shown in Figure 1, the time series generated with different sampling intervals maintain the oscillatory behavior of the original MG dynamics, but the similarity between Figure 1b,c cannot be readily observed by the naked eye.

**Figure 2:**
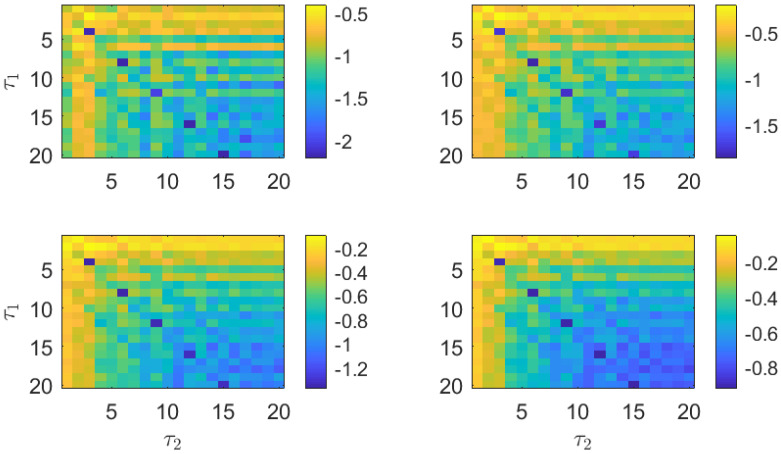
PJSD estimations in logarithmic base 10 scale from two numerical realizations of Mackey–Glass oscillator operating in chaotic regime ($\tau_{S}=30$) at different sampling intervals $t_{b}=18\cdot 10^{2}\Delta t$ and $t_{c}=24\cdot 10^{2}\Delta t$. Order *D* increases from 3 to 6 (from upper left to lower right plots), and lags $\tau_{1}$ and $\tau_{2}$ vary from 1 to 20. Time series of length L=12,500 data points are considered in analysis.

To visualize the properties of the PJSD, we apply this measure to the MG time series presented in Figure 1. For this purpose, the PJSD is computed for the sampled time series $x_{b}$ and $x_{c}$ using varying symbolization lags, $\tau_{1}$ and $\tau_{2}$. The PJSD is expected to capture the similarity between $x_{b}$ and $x_{c}$ for certain ratios of the symbolization lags and sampling intervals. We present the evaluation of the PJSD for the sampled MG time series in Figure 2, computed for several embedding dimensions, *D*, ranging from 3 to 6. In this representation, larger similarities between the analyzed time series correspond to blue colors (PJSD values close to zero, shown on a logarithmic scale). As demonstrated in Figure 2, the PJSD measure successfully recovers the similarity between the MG time series $x_{b}$ and $x_{c}$ for several values of the symbolization lags. The similarity of the time series becomes evident when $\tau_{1}/\tau_{2}=4/3$, a ratio that precisely compensates for the difference in sampling times, $t_{b}/t_{c}=3/4$, from the original MG time series. In other words, the minima of the computed PJSD occur when $\tau_{1}\cdot t_{b}=\tau_{2}\cdot t_{c}$. Consequently, smaller estimated PJSD values are observed not only for $\tau_{1}=4$ and $\tau_{2}=3$, but also for multiples of them: $\tau_{1}=8$ and $\tau_{2}=6$, $\tau_{1}=12$ and $\tau_{2}=9$, and so on. This behavior acts as a double check for identifying the temporal scales under which the time series under analysis are truly similar.

**Figure 3:**
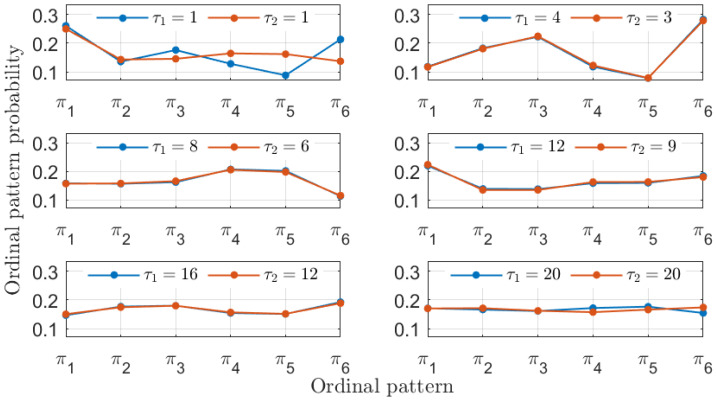
Ordinal pattern probabilities with D=3 for the two numerical realizations of the Mackey–Glass oscillator operating in a chaotic regime ($\tau_{S}=30$) at different sampling intervals $t_{b}=18\cdot 10^{2}\Delta t$ and $t_{c}=24\cdot 10^{2}\Delta t$. Particular choices of the symbolization lags $\tau_{1}$ and $\tau_{2}$ associated with these two signals are considered in each subplot.

**Figure 4:**
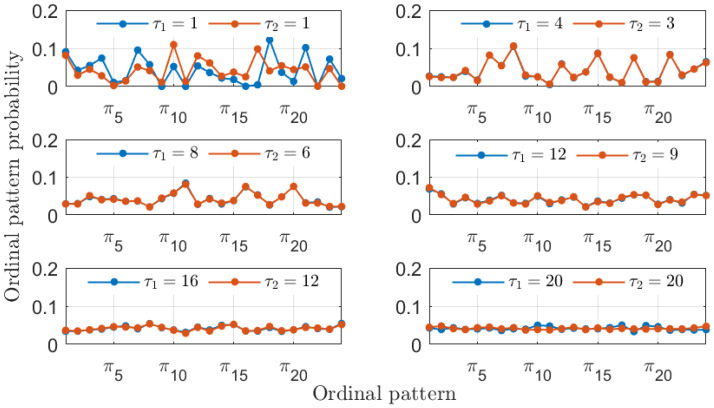
The same as in Figure 3 but with D=4.

In addition to recovering the similarity between the MG time series, the PJSD values shown in Figure 2 exhibit other trends worth discussing. The PJSD values systematically decrease as $\tau_{1}$ and $\tau_{2}$ increase, corresponding to the yellow colors in the top left corners and blue colors in the bottom right corners of the different panels in Figure 2. This trend can be explained by examining the underlying ordinal pattern probabilities upon which the PJSD is computed. For instance, Figure 3 shows the ordinal pattern probabilities for the analyzed MG time series at selected values of $\tau_{1}$ and $\tau_{2}$. When $\tau_{1}/\tau_{2}=4/3$, the measured ordinal pattern probabilities for $x_{b}$ and $x_{c}$ are equal, resulting in PJSD values close to zero. Otherwise, two scenarios arise: either different probabilities are measured when $\tau_{1}$ or $\tau_{2}$ is small, or distributions approaching equiprobability are observed when $\tau_{1}$ and $\tau_{2}$ are both large. This analysis is further supported by examining the ordinal pattern probabilities for higher embedding dimensions *D*, as shown, for example, in Figure 4 for D=4, where the probabilities for $x_{b}$ and $x_{c}$ are equal when $\tau_{1}/\tau_{2}=4/3$. Finally, it can also be observed that the ordinal pattern probabilities for both signals are clearly different if the original sampling intervals are considered, i.e., when $\tau_{1}=\tau_{2}=1$.

**Figure 5:**
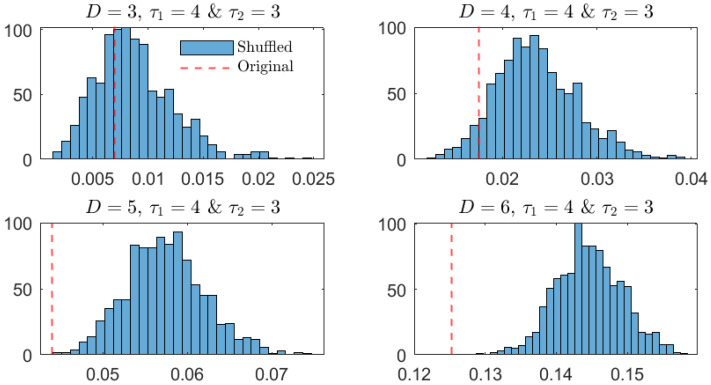
Distribution of 1000 estimated PJSD values from shuffled realizations of original MG time series for different orders *D* when lags $\tau_{1}$ and $\tau_{2}$ are equal to 4 and 3, respectively. Red vertical dashed line indicates estimated PJSD value for the original MG signals.

**Figure 6:**
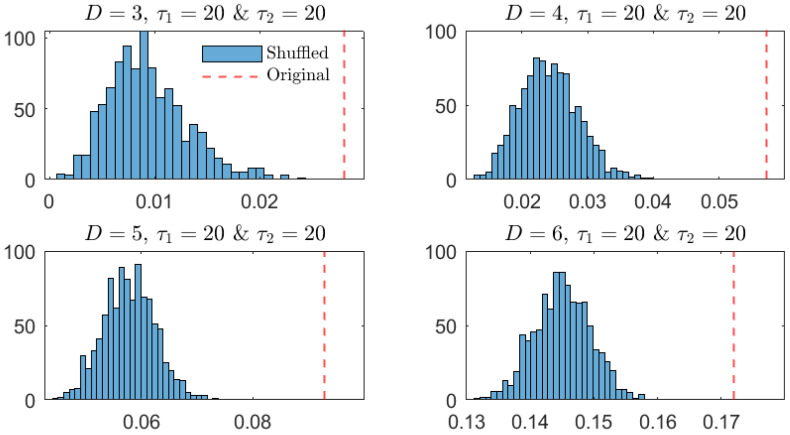
The same as in Figure 5 but for lags $\tau_{1}=20$ and $\tau_{2}=20$.

As additional and more robust confirmation of the similarity between the OPDs for $\tau_{1}=4$ and $\tau_{2}=3$, a surrogate analysis is performed. More precisely, the PJSD of the original time series is compared against the distributions of the estimated PJSD values for 1000 independent shuffled realizations of the original records when the same lags are considered. Just for the sake of comparison, the same analysis is repeated for large values of the lag ($\tau_{1}=20$ and $\tau_{2}=20$). The results obtained are briefly summarized in Figure 5 and Figure 6. On the one hand, when $\tau_{1}=4$ and $\tau_{2}=3$ (Figure 5), it can be concluded that the estimated PJSD value for the original MG time series is significantly lower than those obtained for their shuffled counterparts if larger values of the order D are considered (D=5 and D=6). On the other hand, when $\tau_{1}=20$ and $\tau_{2}=20$ (Figure 6), the estimated PJSD value is significantly higher than those associated with shuffled realizations, independently of the order *D*. Based on these findings, the similarity and dissimilarity between the original MG signals can be robustly concluded in the former and latter cases, respectively.

It is worth remarking here that the running time to estimate the PJSD between the MG signals (L=12,500) for the 400 considered combinations of the lags $\tau_{1}$ and $\tau_{2}$ is around a second. Thus, the proposed multiscale scheme is fast enough, paving the way for real-time analysis. Obviously, the time complexity increases for larger values of the order *D* and for longer time series. Please see Appendix A for further details.

In summary, we have validated the PJSD methodology for identifying similarities between different time series in a controlled numerical example generated from a single parameter set with varying sampling intervals. In the next section, we extend the application of the PJSD methodology to a numerical setting where time series are generated from different parameter sets, as well as to experimental data from a laser system.

## 4. Practical Application: Semiconductor Lasers Subject to Optical Feedback

Semiconductor lasers can exhibit rich dynamical behavior when they are subjected to external perturbations such as optical feedback or optical injection [34]. In particular, semiconductor lasers with optical feedback are typically considered a paradigmatic physical system to observe complex dynamical behavior experimentally [35]. Several time scales are involved in the resulting complex dynamics, and often, the precise characterization is not straightforward due to the nonlinear interactions that occur in the semiconductor laser [26].

The complexity of the intensity emitted by the semiconductor laser subject to optical feedback makes it an ideal case to analyze the validity of the PJSD metric. In the following, we first proceed to describe the system under study, both theoretically and experimentally. We then present the results of the PJSD metric applied to numerical and experimental time series of the laser subject to feedback.

### 4.1. Theoretical Model

A single-mode semiconductor laser subject to moderate optical feedback can typically be described by the following Lang–Kobayashi (LK) equations [36,37],

(5) $$\dot{E}\left(t\right)=\frac{1+i\;\alpha }{2}\;G_{N}\;n\left(t\right)\;E\left(t\right)+\kappa E\left(t-\Lambda \right)e^{-i\omega_{0}\Lambda },$$

(6) $$\dot{n}\left(t\right)=p_{exc}\;J_{\mathrm{th}}-\gamma \;n\left(t\right)-\left[\Gamma +G_{N}\;n\left(t\right)\right]{\left|E\left(t\right)\right|}^{2}\;,$$

where *E* and *n* are the complex electric field amplitude and the carrier number above the threshold, respectively. The equations are normalized such that $P\left(t\right)={\left|E\left(t\right)\right|}^{2}$ is the number of photons. The optical feedback term in the field equation includes the delay time Λ and the feedback rate κ. The other parameters in Equation (5) are the linewidth enhancement factor (α), the differential optical gain ($G_{N}=2.142\cdot 10^{4}\;s^{-1}$) and the laser solitary frequency ($\omega_{0}$). In Equation (6), $p_{exc}$ is the excess pump current over the threshold ($\frac{J}{J_{\mathrm{th}}}-1)$, $J_{\mathrm{th}}=1.552\cdot 10^{17}\;s^{-1}$ denotes the pump threshold current in units of the electron charge, $\gamma =0.909\cdot 10^{9}\;s^{-1}$ is the carrier decay rate, and $\Gamma =0.357\cdot 10^{12}\;s^{-1}$ is the cavity decay rate. The parameter values were chosen according to Refs. [34,37], except for α=3, $\Lambda =10\;{\mathrm{ns}}^{-1}$, in order to obtain dynamical behavior similar to the experimental behavior [38].

The relaxation oscillation (RO) frequency is the natural resonance of the semiconductor laser, which results from light–matter interactions. In this model, the RO is given by $f_{RO}=\frac{1}{2\pi }\sqrt{G_{N}p_{exc}J_{th}}$. In turn, the optical feedback induces a frequency shift in the emitted optical frequency, where the maximum feedback-induced frequency shift is $\Delta f_{fb}∼\alpha \kappa /2\pi$. As shown in Ref. [38], similar dynamics can be observed for different laser and feedback conditions as long as the ratio between $f_{RO}$ and $\Delta f_{fb}$ is kept constant. Here, similarity refers to the phenomenon of observing laser time series with equivalent statistical properties but with different time scales. We control the ratio between $f_{RO}$ and $\Delta f_{fb}$ by changing the values of $p_{exc}$ and κ, which are readily accessible to the experimentalist. To be precise, the time scale of the delay time Λ will also need to be adjusted by a factor of $\sqrt{p_{exc}}$ to achieve similar laser dynamics.

### 4.2. Description of Experimental Setup

**Figure 7:**
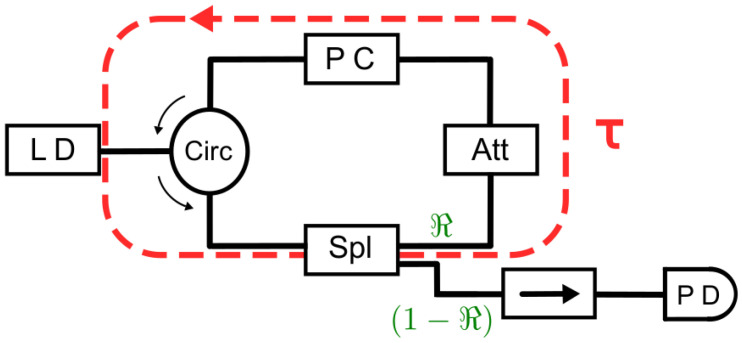
Scheme of experimental setup to study feedback dynamics. LD: laser diode; Circ: optical circulator; PC: polarization controller; Att: optical attenuator; Spl: one-by-two intensity splitter with ℜ=0.95 and (1−ℜ)=0.05 splitting ratios; →: optical isolator; and PD: photodiode.

Figure 7 depicts the scheme of the experimental setup, achieved by employing standard fiber-based telecommunication components. The semiconductor laser diode in the experimental setup has an emission wavelength around 1550 nm. This laser emits in a single longitudinal mode, with side-mode suppression ratios larger than 40 dB, and has a threshold current of $I_{th}$ = 12.08 mA at a working temperature of 22 °C. The dashed red line in Figure 7 encloses the external cavity of round-trip time Λ, which depends on the length of the fiber components of the external cavity feedback loop. Such a fiber-optic external cavity has characteristic round-trip times of 10 to 100 ns. In the external cavity loop, a maximum feedback rate of $\kappa_{max}$≃ 70 ns^−1^ was estimated [38]. Time series were acquired using a photodiode with a 12.5 GHz bandwidth and a 16 GHz analog bandwidth oscilloscope with a sampling rate of 40 Gigasamples/s.

### 4.3. Numerical Results

Motivated by the experimental settings, we consider two numerically generated time series that have a constant sampling rate but are generated using different laser and feedback parameters. Similarity of two laser time series, *A* and *B*, is expected to be found when $\kappa_{A}/\sqrt{p_{excA}}=\kappa_{B}/\sqrt{p_{excB}}$ and $\sqrt{p_{excA}}\cdot \Lambda_{A}=\sqrt{p_{excB}}\cdot \Lambda_{B}$ are fulfilled [38]. In particular, we choose, for illustrative purposes, the following two parameter sets: $p_{excA}$ = 0.25, $\kappa_{A}=50\;{\mathrm{ns}}^{-1}$, $\Lambda_{A}=11.2\;\mathrm{ns}$, and $p_{excB}$ = 1, $\kappa_{B}=100\;{\mathrm{ns}}^{-1}$, $\Lambda_{B}=5.6\;\mathrm{ns}$, respectively. As a result, the two numerically generated time series should be equivalent for sampling intervals that correspond to a ratio 2 to 1, i.e., $\sqrt{1/p_{excA}}$ to $\sqrt{1/p_{excB}}$.

**Figure 8:**
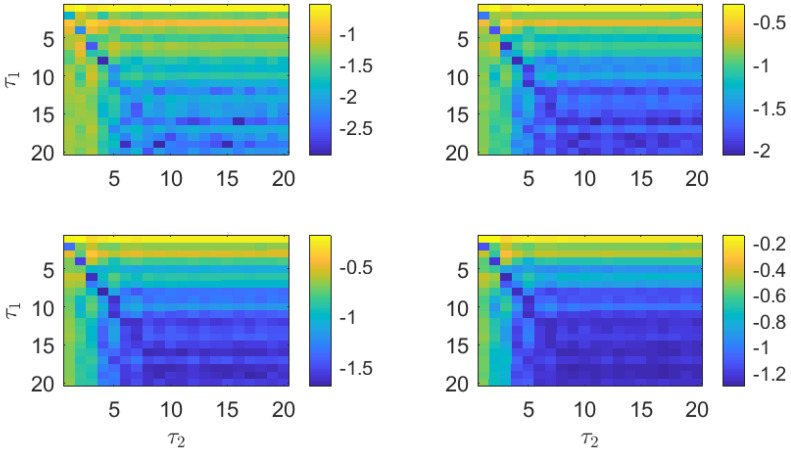
PJSD estimations in logarithmic base 10 scale from two numerical realizations of LK equations ($L=10^{5}$ data points) at different time scales but with equivalent statistical properties. Order *D* increases from 3 to 6 (from upper left to lower right plots), and lags $\tau_{1}$ and $\tau_{2}$ vary from 1 to 20. Numerical laser time series are subsampled to 10/Γ≃ 28 ps prior to analysis.

The estimations of the PJSD for the two numerically generated laser time series are presented in Figure 8. Independently of the embedding dimension *D*, low values of the PJSD are obtained when the symbolization lags $\tau_{1}$ and $\tau_{2}$ follow the 2-to-1 ratio. Similarity of the compared laser time series is recovered for several combinations of the symbolization lags, including $\tau_{1}=4$ and $\tau_{2}=2$; $\tau_{1}=6$ and $\tau_{2}=3$; and $\tau_{1}=8$ and $\tau_{2}=4$.

**Figure 9:**
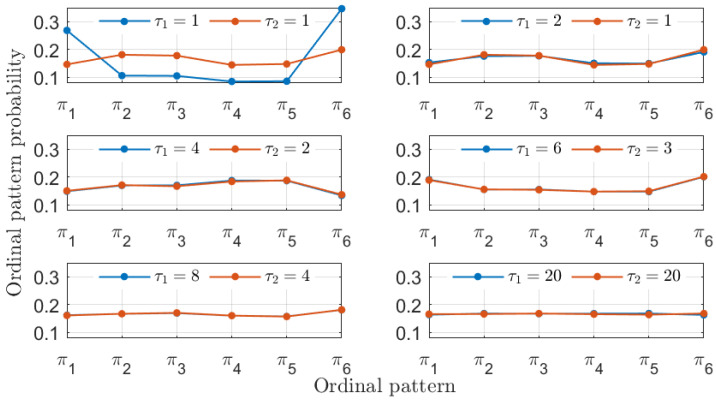
Ordinal pattern probabilities with D=3 for the two numerical simulations of the LK equations. Particular choices of the symbolization lags $\tau_{1}$ and $\tau_{2}$ associated with these two signals are considered in each subplot.

In agreement with the results presented for the MG time series in Figure 2, large PJSD values (yellow colors) tend to appear for small values of the symbolization lags, and low PJSD values (blue colors) appear for large values of the symbolization lags. As shown in Figure 9, low values of the PSJD metric can be obtained either when the ordinal pattern probabilities are computed following a 2-to-1 ratio or when the symbolization lags are large, obtaining quasi-equiprobable distributions.

**Figure 10:**
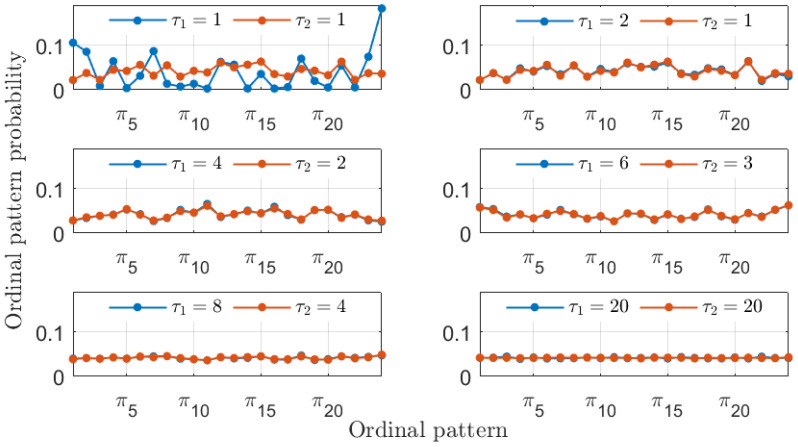
The same as in Figure 9 but with D=4.

The ordinal pattern probabilities computed using D=3 are relatively simple. It is also interesting to observe the probabilities obtained using a larger embedding dimension. Figure 10 presents the ordinal pattern probabilities computed using D=4. In this case, the similarity of the numerical laser time series is recovered for the proper 2-to-1 ratio of the symbolization lags, although the observed probabilities are significantly more involved.

### 4.4. Experimental Results

We now analyze two experimental time series of a semiconductor laser subject to optical feedback. Identifying the similarity of experimental time series is a challenging task, as the data may contain noise and other non-idealities. Considering the dynamical study of this laser system, we can identifying similarity when the two laser time series *A* and *B* fulfill the conditions $\kappa_{A}/\sqrt{p_{excA}}=\kappa_{B}/\sqrt{p_{excB}}$ and $\sqrt{p_{excA}}\cdot \Lambda_{A}=\sqrt{p_{excB}}\cdot \Lambda_{B}$. In this case, we analyze time series with the parameter sets $p_{excA}$ = 0.57 ($\sqrt{p_{excA}}≃$ 3/4), $\Lambda_{A}=99.95\;\mathrm{ns}$ and $p_{excB}$ = 1, $\Lambda_{B}=75.18\;\mathrm{ns}$ ($\Lambda_{B}≃3/4\Lambda_{A}$), respectively. Accordingly, the feedback strengths are set to $\kappa_{B}=4/3\kappa_{A}$ such that the same ratio between $f_{RO}$ and $\Delta f_{fb}$ is maintained for the two experimental laser time series.

**Figure 11:**
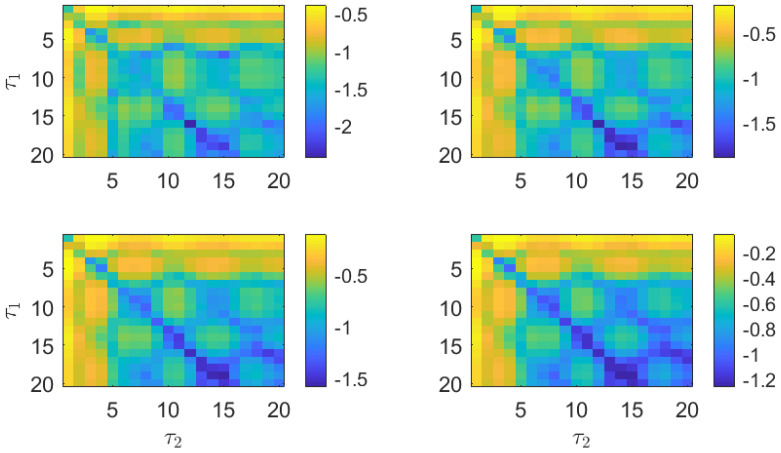
PJSD estimations in logarithmic base 10 scale from two experimentally obtained signals ($L=10^{5}$ data points) with dynamical behaviors similar to numerical counterparts. Order *D* increases from 3 to 6 (from upper left to lower right plots), and lags $\tau_{1}$ and $\tau_{2}$ vary from 1 to 20. Experimental laser time series are acquired with sampling interval of 25 ps for analysis.

We present the PJSD estimations of the two experimental laser time series in Figure 11. A slanted blue line, corresponding to a 4-to-3 ratio between the symbolization lags $\tau_{1}$ and $\tau_{2}$, can be observed for all embedding dimensions. This result implies that the same dynamical features can be reproduced at different time scales when the laser and feedback parameters are properly adjusted. Since semiconductor lasers can be used in optical communications for secure key distribution [39,40,41], similarity could be exploited, e.g., to transmit secure keys at different rates.

**Figure 12:**
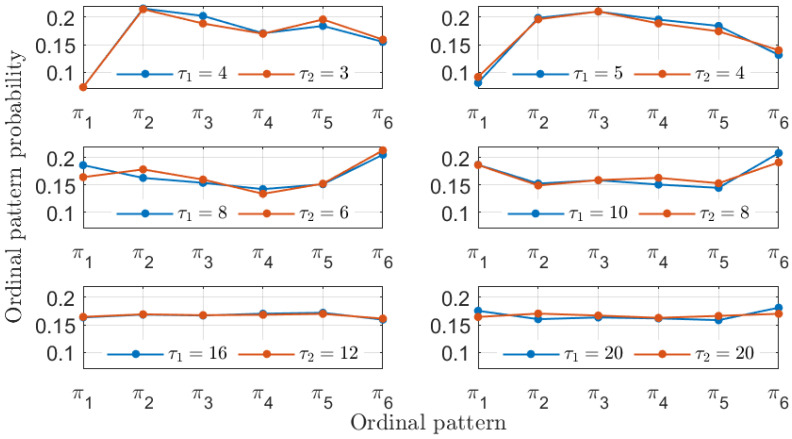
Ordinal patterns probabilities with D=3 for the two experimental signals. Particular choices of the symbolization lags $\tau_{1}$ and $\tau_{2}$ associated with these two signals are considered in each subplot.

**Figure 13:**
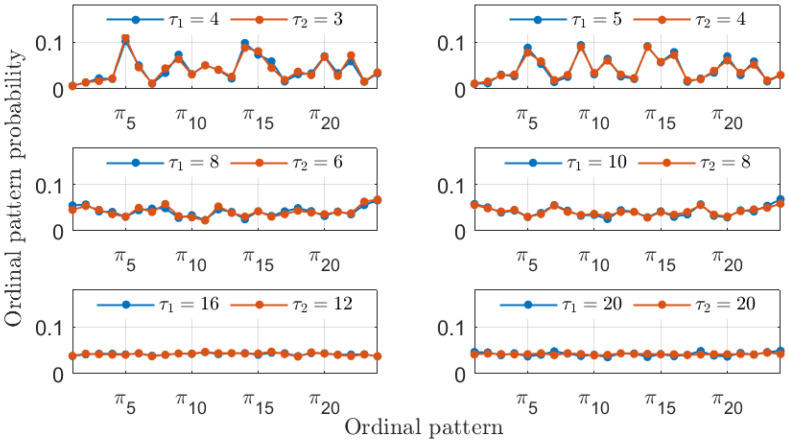
The same as in Figure 12 but with D=4.

The similarity between the two experimental laser time series is further evidenced by the ordinal pattern probabilities displayed in Figure 12 and Figure 13. Here, given the limited experimental precision, the 4-to-3 ratio for the similarity is not exact, and the 5-to-4 ratio also yields similar ordinal pattern probabilities. The temporal precision of identifying similarity is bounded by the sampling interval of 25 ps corresponding to the acquisition oscilloscope. Nevertheless, the existence of similarity between the two experimental laser time series can be readily identified given the results in Figure 11.

## 5. Conclusions

The ability of the PJSD to characterize the ordinal similarity of two time series at different temporal scales has been carefully analyzed in this work. Through numerical and experimental analyses, it has been shown that the PJSD offers a simple and flexible approach for identifying the sampling rates that minimize the distance between two arbitrary sequences from an ordinal perspective. This finding stands in stark contrast to what happens with other popular similarity measures, such as dynamic time warping, which show reduced performance when dealing with this issue.

Given its versatility, robustness to noise and outliers, and invariance under data scaling, it is reasonable to predict that the proposed multiscale PJSD approach could be of utility in analyses of real-world data from heterogeneous scientific fields. We encourage interested researchers and practitioners to implement this tool in order to confirm this hypothesis.

## Figures and Tables

**Figure A1:**
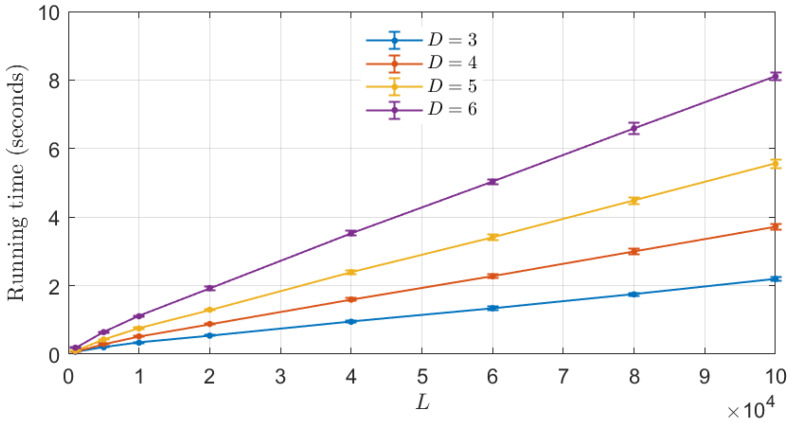
Running time to estimate the multiscale PJSD as a function of the time series length *L* for different orders *D*. The mean and standard deviation (presented as error bars) of the running time, from 100 independent estimations of the PJSD between two Gaussian white noise numerical realizations with order *D* and lags $\tau_{1}$ and $\tau_{2}$ between 1 and 20 (400 combinations), are plotted.

## Data Availability

The raw data supporting the conclusions of this article will be made available by the authors on request.

## Abbreviations

The following abbreviations are used in this manuscript:

PJSD	Permutation Jensen–Shannon distance
JSD	Jensen–Shannon divergence
BP	Bandt and Pompe
OPD	Ordinal probability distribution
MG	Mackey–Glass
LK	Lang–Kobayashi
RO	Relaxation oscillation

## Appendix A. Computational Time

With the aim of obtaining a better handle on the computational cost of the multiscale scheme proposed in this work, the time necessary to perform this multiscale analysis by running a self-made MATLAB (Version 9.5, R2018b) script was calculated. Being more precise, a pair of arbitrary time series (Gaussian white noise in the following analysis) of length *L* were generated, and then, the running time to estimate the PJSD between these two signals for the 400 combinations of lags $\tau_{1}$ and $\tau_{2}$ with $1\leq \tau_{1},\tau_{2}\leq 20$ were obtained. This analysis was repeated for 100 independent pairs of simulations, varying the time series length *L* ($L\in \{10^{3},5\cdot 10^{3},10^{4},2\cdot 10^{4},4\cdot 10^{4},6\cdot 10^{4},8\cdot 10^{4},10^{5}\}$) and the order *D* (D∈{3,4,5,6}). The results obtained for the running times (in seconds) as a function of length *L* for the different orders *D* are illustrated in Figure A1. The mean and standard deviation (presented as error bars), from the 100 independent pairs of numerical simulations, were plotted. The time complexity increases linearly with the time series length *L*, with a slope that depends on the order *D*. Obviously, higher computational costs are observed as *D* increases, since the number of possible ordinal patterns is larger. It is important to highlight that even in the worst case, i.e., D=6 and $L=10^{5}$, the average time required to estimate the PJSD for the 400 combinations of lags $\tau_{1}$ and $\tau_{2}$ is around 8 s. Consequently, the proposed methodology is fast enough and can be implemented for the analysis of large databases.
